# Consumption of Terpenoids-Rich *Padina pavonia* Extract Attenuates Hyperglycemia, Insulin Resistance and Oxidative Stress, and Upregulates PPARγ in a Rat Model of Type 2 Diabetes

**DOI:** 10.3390/antiox9010022

**Published:** 2019-12-26

**Authors:** Mousa O. Germoush, Hassan A. Elgebaly, Sherif Hassan, Emadeldin M. Kamel, May Bin-Jumah, Ayman M. Mahmoud

**Affiliations:** 1 Biology Department, College of Science, Jouf University, Sakaka 2014, Saudi Arabia; germoush@ju.edu.sa (M.O.G.); hagebaly@ju.edu.sa (H.A.E.); 2 Botany and Microbiology Department, Faculty of Science, Beni-Suef University, Beni-Suef 62514, Egypt; sherif.mohamed@science.bsu.edu.eg; 3 Chemistry Department, Faculty of Science, Beni-Suef University, Beni-Suef 62514, Egypt; emad.abdelhameed@science.bsu.edu.eg; 4 Biology Department, College of Science, Princess Nourah bint Abdulrahman University, Riyadh 84428, Saudi Arabia; mnbinjumah@pnu.edu.sa; 5 Physiology Division, Zoology Department, Faculty of Science, Beni-Suef University, Beni-Suef 62514, Egypt

**Keywords:** diabetes, terpenes, seaweeds, oxidative stress, PPARs

## Abstract

Seaweeds are rich in structurally diverse bioactive compounds with promising therapeutic effects. This study aimed to isolate and identify terpenes from the brown alga *Padina pavonia* and to investigate its antidiabetic activity, pointing to the possible involvement of peroxisome proliferator-activated receptor (PPAR)γ. Type 2 diabetes was induced by feeding rats a high fat diet (HFD) for 4 weeks followed by injection of 35 mg/kg streptozotocin (STZ). The diabetic rats received *P. pavonia* extract (PPE; 50, 100 and 200 mg/kg) for 4 weeks and samples were collected for analyses. HFD/STZ-induced rats showed hyperglycemia, dyslipidemia, impaired glucose tolerance, decreased insulin, and increased HbA1c and HOMA-IR. PPE ameliorated hyperglycemia and dyslipidemia, and improved glucose tolerance and insulin sensitivity in diabetic rats. Treatment with PPE increased hepatic hexokinase activity and glycogen, suppressed glucose-6-phosphatase, fructose-1,6-biphosphatase, and glycogen phosphorylase, and attenuated oxidative stress, inflammation, and liver injury and lipid infiltration in HFD/STZ-induced rats. In addition, PPE boosted antioxidants and upregulated PPARγ gene and protein expression in the liver of diabetic rats. Phytochemical investigation resulted in the isolation of six terpenes from PPE and in silico analysis revealed their binding affinity toward PPARγ. In conclusion, *P. pavonia*-derived terpenes attenuated hyperglycemia, dyslipidemia, oxidative stress, and inflammation, and improved insulin sensitivity and carbohydrate metabolism in type 2 diabetic rats. These beneficial effects are mediated via PPARγ activation. However, further studies to explore the exact mechanisms underlying the antidiabetic effect of PPE are recommended.

## 1. Introduction

Diabetes mellitus (DM) is a metabolic disease characterized by chronic hyperglycemia resulting from impaired insulin secretion and/or action. It is a major global health concern rising to an alarming epidemic level. The number of people with diabetes was estimated to be 451 million in 2017 and this number is expected to reach 693 million by 2045 [1]. Type 2 DM (T2DM) is the most prevalent form of the disease that affect more than 380 million people worldwide [1,2]. T2DM is characterized by insulin resistance (IR) and progressive loss of β-cell function, resulting in hyperglycemia [2]. If not tightly controlled, hyperglycemia can lead to organ damage and serious complications, including retinopathy, neuropathy, and nephropathy [3]. In addition, diabetic patients have increased risk of developing different liver diseases [4]. IR is associated with impaired insulin action and signaling in the muscle, liver, and adipose tissue [5]. It increases the demand to insulin which could not be met by the β-cells, leading to decreased insulin release and gradual destruction of these cells [6]. Hyperlipidemia is another common characteristic feature of T2DM [7]. Elevated circulating lipids and the accumulation of lipids within insulin-responsive tissues are clearly associated with IR [8].

Oxidative stress and inflammation are associated with the development of T2DM. Oxidative stress in DM occurs as a result of elevated glucose and/or fatty acid oxidation mediated excessive production of reactive oxygen species (ROS). Excess ROS can reduce insulin release from β-cells, impair insulin sensitivity and signaling in insulin-responsive tissues, and influence the development of diabetic complications [9]. In addition, ROS can alter cell function and induce lipid peroxidation (LPO), inflammation, and apoptosis in the pancreas, kidney, liver, nerves, and vasculature [10]. Clinical studies have demonstrated increased LPO markers in the plasma and erythrocytes of patients with T2DM [11,12]. Besides oxidative stress, ROS can activate nuclear factor-kappaB (NF-κB) and the release of pro-inflammatory cytokines. In this context, increased circulating levels of interleukin (IL)-6 and tumor necrosis factor (TNF)-α have been demonstrated in T2DM in human patients [13] as well as rodents [14,15]. These cytokines impair insulin signaling, glucose tolerance, and peripheral glucose uptake, and provoke IR, lipolysis, and hepatic glucose production [16,17,18,19,20]. Therefore, attenuation of hyperglycemia and its associated oxidative stress and inflammation represents an effective strategy to ameliorate IR.

Peroxisome proliferator-activated receptors (PPARs) are ligand-inducible transcription factors that regulate the expression of multiple genes involved in adipogenesis, metabolism, and inflammation. A number of metabolic pathways implicated in T2DM, metabolic syndrome, and other metabolic diseases are also regulated by PPARs [21]. PPARγ plays a key role in the regulation of insulin sensitivity, glucose and lipid metabolism, and other metabolic processes. PPARγ agonists have been demonstrated to increase insulin sensitivity, lower plasma glucose and lipids, ameliorate hepatic, cardiac and muscular lipid accumulation, suppress inflammation and reduce ROS generation [22,23]. Activation of PPARγ has also been associated with suppressed oxidative stress and inflammation in animal models of hepatic fibrosis, injury, and carcinogenesis [24,25,26]. Therefore, activation of PPARγ is an effective strategy against the development and progression of various metabolic diseases.

Marine algae represent a rich reservoir of bioactive compounds that might represent promising leads in the development of effective therapeutic agents [27]. *Padina pavonia* L. is a brown seaweed containing free fatty acids, phenols, terpenoids, aromatic esters, and sulfated polysaccharides [28,29]. *P. pavonia* exhibited anti-bacterial, anti-coagulant [28,29], antioxidant, and anti-inflammatory activities [30,31]. We have previously reported that the hepatoprotective and anti-carcinogenic effects of *P. pavonia* ethanolic extract were associated with attenuation of oxidative stress and inflammation and upregulation of PPARγ in mice [31,32]. Given the promising anti-inflammatory and other therapeutic effects of marine algae derived terpenoids [33], we aimed to isolate and identify terpenes from *P. pavonia* and to study their binding affinity toward PPARγ. In addition, we investigated the potential of a terpenoid rich fraction of *P. pavonia* to ameliorate hyperglycemia, IR, and oxidative stress in a rat model of T2DM, pointing to the possible involvement of PPARγ.

## 2. Materials and Methods

### 2.1. Collection of P. pavonia, Extraction, and Isolation

#### 2.1.1. General Experimental Procedures

^1^H (400 MHz) and ^13^C (100 MHz) NMR were recorded in CDCl3 on a Varian Unity Inova instrument using TMS as the internal standard. Chemical shifts (δ) were expressed in ppm and coupling constants (J) were reported in Hz. Optical rotations were measured using Perkin-Elmer 341 polarimeter and Shimadzu UV–vis 160i spectrophotometer was used for UV measurements. HREIMS and EIMS mass spectroscopic analysis were measured on a Finnigan MAT TSQ 700 mass spectrometer. IR spectra were recorded on KBr pellets on a Shimadzu FTIR-8400 instrument.

#### 2.1.2. Extraction and Isolation

*P. pavonia* was collected from the Red Sea shore at Kiyal Valley and Ras Al Sheikh Humaid, Tabuk province (Saudi Arabia). The samples were washed with sea water followed by tap water and distilled water to remove sand and salt, and then air-dried in shade. The dried algal material (1.5 kg) was powdered and exhaustively extracted with dichloromethane (DCM) (15 × 4 L, each for 72 h). The solvent was removed at a temperature less than 40 °C and reduced pressure till complete dryness to yield a brownish residue (32 g). The DCM extract was suspended in 1 L H_2_O, then successively extracted with ethyl acetate (EtOAc; 1 L × 3) and *n*-butanol (1 L × 3) to yield EtOAc fraction (13 g) and *n*-butanol fraction (9 g). The EtOAc fraction was applied to silica gel column chromatography (CC) (200–300 mesh, 3.5 × 100 cm, 230 g), and gradient elution started with *n*-hexane followed by *n*-hexane/EtOAc mixtures of increasing polarity polarities (10:0 → 0:10 *v*/*v*) to afford 38 fractions. The eluted fractions were monitored by thin layer chromatography (TLC) and combined based on their TLC behavior to produce a total of 17 fractions (E1–E17). Fraction E6 was re-chromatographed over silica gel cc (25 × 2 cm) and eluted chloroform (CHCl_3_)-methanol (MeOH) (10:1 → 5:1) to yield 7 sub-fractions (E6.1–E6.7). Sub-fraction E6.3 was purified using ODS cc (1 × 20 cm) and eluted with MeOH to produce the purified compound **3** (16 mg). Fraction E10 was applied to silica gel cc (35 × 2 cm) using *n*-hexane-EtOAc (5:1 → 1:1) as an eluent to give rise to nine sub-fractions (E10.1–E10.9). Sub-fraction E10.3 was further purified over silica gel cc (1 × 15 cm) eluted with *n*-hexane-EtOAc (2:1) to produce compound **4** (13 mg). Sub-fraction E10.8 was re-chromatographed using silica gel cc (1.5 × 20 cm) using *n*-hexane-CHCl_3_-MeOH (7:5:1) to produce five sub-fractions (E10.8.1–E10.8.5). Sub-fraction E10.8.3 was purified using ODS cc (1 × 15 cm) and eluted with MeOH-H_2_O (2:1) to produce the purified compound **2** (11 mg). Fraction E12 was subjected to silica gel cc (1.5 × 50 cm) eluted with *n*-hexane-EtOAc ((5:1 → 1:5) to produce five fractions (E12.1–E12.5). The sub-fraction E12.2 was purified over Sephadex LH-20 CC (1 × 15 cm) and eluted with acetone to afford compound **1** (18 mg), whereas the sub-fraction E12.5 was placed on the top of a silica gel cc (1.5 × 25 cm) eluted with petroleum ether/acetone system (5:1 → 1:5) to produce five sub-fractions (E12.5.1–E12.5.5). Compound **5** (15 mg) was obtained from sub-fraction E12.5.3 after purification over RP-18 gel cc using MeOH/H_2_O (9.5:0.5) as an eluent. Sub-fraction E12.5.4 was further purified over ODS cc (1 × 15 cm) using MeOH-H_2_O (2:1) to afford compound **6** (16 mg).

##### *3α-Hydroxy-5,6-epoxy-7-megastigmen-9-one* (**1**)

Colorless needles, [α]_D_^25^: −97.4° (MeOH, *c* 1.4); UV (MeOH) nm λ_max_ (log ε): 317 (2.94), 295 (2.89), 229 (4.12); IR (KBr) ν_max_ cm^−1^: 3405, 2965, 2922, 1701, 1384, 1299, 1105, 1004, 967; HR-ESI-MS (positive ion mode): *m/z* 224.1418 [M + H]^+^ (calcd for C_13_H_20_O_3_, 224.1412); for ^1^H NMR (CDCl_3_, 400 MHz) and ^13^C NMR (CDCl_3_, 100 MHz) data, see Table 1.

##### *(+)-Dehydrovomifoliol* (**2**)

Colorless needles, [α]_D_^25^: 260° (MeOH, *c* 0.2); UV (MeOH) nm λ_max_ (log ε): 235 (2.45), 202 (2.47); IR (KBr) ν_max_ cm^−1^: 3425, 2981, 1698, 1311, 1286, 1117, 1013; HR-ESI-MS (positive ion mode): *m/z* 222.1261 [M+H]^+^ (calcd for C_13_H_18_O_3_, 224.1256); for ^1^H NMR (CDCl_3_, 400 MHz) and ^13^C NMR (CDCl_3_, 100 MHz) data, see Table 1.

##### *Loliolide* (**3**)

Colorless needles, [α]_D_^25^: −96.8° (CHCl_3_, *c* 0.95); UV (MeOH) nm λ_max_ (log ε): 214 (4.13); IR (KBr) ν_max_ cm^−1^: 3380, 2945, 1719, 1618, 1277, 1286; HR-ESI-MS (positive ion mode): *m/z* 197.1109 [M+H]^+^ (calcd for C_11_H_16_O_3_, 197.1099); for ^1^H NMR (CDCl_3_, 400 MHz) and ^13^C NMR (CDCl_3_, 100 MHz) data, see Table 1.

##### *(6R,7E,9R)-9-hydroxy-4,7-megastigmadien-3-one* (**4**)

Colorless oil, [α]_D_^25^: 157° (CHCl_3_, *c* 0.14); IR (KBr) ν_max_ cm^−1^: 3410, 2945, 1701, 1625, 1277, 982; EI/MS *m/z* 208 [M]^+^; for ^1^H NMR (CDCl_3_, 400 MHz) and ^13^C NMR (CDCl_3_, 100 MHz) data, see Table 2.

##### *Petasol* (**5**)

Colorless gum, [α]_D_^25^: 92° (CHCl_3_, *c* 1.0); UV (MeOH) nm λ_max_ (log ε): 237 (3.63), 204 (4.14); IR (KBr) ν_max_ cm^−1^: 3427, 2908, 1698, 1617, 1209, 991; EI/MS *m/z* 234 [M]^+^; for ^1^H NMR (CDCl_3_, 400 MHz) and ^13^C NMR (CDCl_3_, 100 MHz) data, see Table 2.

##### *Oplodiol (7-Eudesmene-1b,4b-diol)* (**6**)

Colorless powder, [α]_D_^25^: −9° (EtOH, *c* 0.1); EI/MS *m/z* 238[M]^+^; IR (KBr) ν_max_ cm^−1^: 3447, 2936, 1615, 1209; for ^1^H NMR (CDCl_3_, 400 MHz) and ^13^C NMR (CDCl_3_, 100 MHz) data, see Table 2.

### 2.2. Animals and Treatments

Male Wistar rats (160–180 g) obtained from Vacsera (Giza, Egypt) were included in this investigation. The animals were housed under standard conditions (23 ± 2 °C and 50–60% humidity) and were given a free access to food and water. The experimental protocol and treatments were approved by the Animal Care and Use Committee.

The rats were allocated into seven groups, two groups were fed normal chow diet and five groups received high fat diet (HFD; 58% fat, 25% protein, and 17% carbohydrate). After 4 weeks, HFD-fed rat received a single intraperitoneal (i.p.) injection of streptozotocin (STZ; 35 mg/kg; Sigma, St. Louis, MO, USA) dissolved in citrate buffer (pH 4.5), and the control rats received i.p. injection of citrate buffer. Seven days after STZ injection, overnight fasted rats received glucose (3 g/kg) orally and blood glucose levels were determined after 2 h. Rats with blood glucose levels ≥ 250 mg/dL were selected to investigate the anti-diabetic effect of the EtOAc fraction of *P. pavonia*.

The rats were allocated into the following groups (*n* = 6):

Group I (Control): received 0.5% carboxymethyl cellulose (CMC) orally for 4 weeks.

Group II (200 mg/kg PPE): received 200 mg/kg *P. pavonia* extract (PPE) dissolved in 0.5% CMC orally for 4 weeks.

Group III (HFD/STZ): received 0.5% CMC orally for 4 weeks.

Group IV (HFD/STZ + 50 mg/kg PPE): received 50 mg/kg PPE dissolved in 0.5% CMC orally for 4 weeks.

Group V (HFD/STZ + 100 mg/kg PPE): received 100 mg/kg PPE dissolved in 0.5% CMC orally for 4 weeks.

Group VI (HFD/STZ + 200 mg/kg PPE): received 200 mg/kg PPE dissolved in 0.5% CMC orally for 4 weeks.

Group VII (HFD/STZ + PIO): received 10 mg/kg pioglitazone (PIO; a PPARγ agonist) [34] dissolved in 0.5% CMC orally for 4 weeks.

After the treatment period, overnight fasted rats were sacrificed under anesthesia. A blood sample from each rat was collected in a tube containing EDTA for glycated hemoglobin (HBA1c) estimation, whereas other blood was collected to prepare serum. The animals were immediately dissected, and the liver was removed, weighed, and a 10% *w*/*v* homogenate was prepared in cold phosphate buffered saline (PBS). Following centrifugation, the clear supernatant was collected for the assay of ROS, LPO, nitric oxide (NO), reduced glutathione (GSH), superoxide dismutase (SOD), and catalase (CAT). Other samples were collected on 10% neutral buffered formalin for histological investigation, whereas others were stored at −80°C.

### 2.3. Oral Glucose Tolerance Test (OGTT)

To investigate the effect of PPE on glucose tolerance, blood samples were collected from the lateral tail vein of overnight fasted rats. Other blood samples were collected at 30, 60, 90, and 120 min following the oral administration of 3 g/kg glucose solution. Glucose was assayed using Spinreact reagent kit (Girona, Spain) according to the method of Trinder [35].

### 2.4. Determination of HBA1c, Insulin, and HOMA-IR

HbA1c and insulin were determined using Biosystems (Barcelona, Spain) and RayBiotech (Peachtree Corners, GA, USA) assay kits, respectively. Homeostasis model of insulin resistance (HOMA-IR) [36] was calculated using the following equation:

(1) $$\mathrm{HOMA}-\mathrm{IR}=\mathrm{Fasting}\;\mathrm{insulin}\;\left(\frac{µU}{\mathrm{ml}}\right)\times \;\mathrm{Fasting}\;\mathrm{glucose}\;\left(\frac{\mathrm{mmol}}{L}\right)/22.5$$

### 2.5. Determination of Glycogen and Glucose-Metabolizing Enzymes

Liver glycogen content was determined according to the method of Seifter et al. [37]. The activities of hexokinase [38], glucose-6-phosphatase (G-6-Pase) [39], fructose-1,6-biphosphatase (F-1,6-BPase) [40], and glycogen phosphorylase [41] were determined in the liver homogenate of control and diabetic animals. Inorganic phosphorus produced during the assay of these enzymes was measured according to the method of Fiske and Subbarow [42] using Spinreact (Girona, Spain) reagent kit.

### 2.6. Determination of Serum and Liver Lipids

Serum triglyceride (TG), total cholesterol (TC), and high-density lipoprotein cholesterol (HDL-C) were assayed using Spinreact (Girona, Spain) kits according to the manufacturer’s instructions. Very low-density lipoprotein cholesterol (vLDL-C) and LDL-C were calculated using the following equations:

(2) vLDL.C=TG/5

(3) LDL.C=TC−(HDL.C+vLDL.C)

Atherogenic index of plasma (AIP) was calculated as follows [43]:

(4) $$\mathrm{AIP}=\mathrm{Log}10\;\left(\frac{\mathrm{TG}}{\mathrm{HDL}.C}\right)$$

To determine the TC and TG levels in the liver, total lipids were extracted by chloroform:methanol mixture (2:1, *v*/*v*) following the method of Folch et al. [44]. Briefly, 100 mg liver was homogenized in 500 μL PBS and the homogenate was centrifuged at 10,000 rpm for 10 min. The supernatant was transferred to a new tube and mixed with 1 mL chloroform/methanol followed by sonication for 30 min. The chloroform phase was collected, dried, and 1 mL isopropanol was added for reconstitution. TG and TC were determined using Spinreact (Girona, Spain) kits.

### 2.7. Determination of Aminotransferases and Cytokines

Serum alanine aminotransferase (ALT) and aspartate aminotransferase (AST) were determined using Spinreact (Girona, Spain) reagent kits. TNF-α and IL-6 were assayed using specific ELISA kits (R&D Systems, Minneapolis, MN, USA).

### 2.8. Determination of Oxidative Stress Markers and Antioxidants

ROS was assayed using 2′,7′-dichlorodihydrofluorescein diacetate (H_2_DCF-DA) as previously described [45]. LPO [46], NO [47], GSH [48], SOD [49], and CAT [50] were determined in the liver homogenate of control and diabetic rats.

### 2.9. Histology

Samples from the liver were fixed for 24 h and then processed for dehydration and embedding in paraffin wax. Five micrometer sections were cut, deparaffinized, rehydrated, and stained with hematoxylin and eosin (H&E). Thereafter, the sections were examined using a light microscope.

### 2.10. PPARγ Expression

The effect of PPE and HFD/STZ on liver PPARγ was determined by qRT-PCR and Western blotting as previously reported [26,51]. Briefly, RNA was isolated using TRIzol reagent (Invitrogen, Waltham, MA, USA) and treated with RNase-free DNase (Qiagen, Germany). The purified RNA was quantified on a nanodrop and samples with A260/A280 nm >1.7 were selected for reverse transcription. cDNA was amplified using SYBR Green master mix and the following primers; PPARγ forward: 5′-GGACGCTGAAGAAGAGACCTG-3′, PPARγ reverse: 5′-CCGGGTCCTGTCTGAGTATG-3′, β-actin forward: 5′-AGGAGTACGATGAGTCCGGC-3′ and β-actin reverse: 5′-CGCAGCTCAGTAACAGTCCG-3′. The obtained data were analyzed by the 2^−ΔΔCt^ method [52]. For Western blotting, liver samples were homogenized in RIPA buffer with proteinase inhibitors and protein content was estimated using Bradford reagent [53]. Forty microgram protein was electrophoresed on 10% SDS/PAGE and transferred to nitrocellulose membrane. After blocking in 5% skimmed milk in tris buffered saline/tween 20 (TBST) for 1 h at room temperature (RT), the membrane was probed with anti-PPARγ and anti-β-actin (Novus Biologicals, USA) overnight at 4 °C. The blots were washed with TBST and incubated with the secondary antibodies for 1 h at RT. After washing with TBST, the membrane was developed using enhanced chemiluminescence detection kit (BIO-RAD, Philadelphia, PA, USA). The obtained bands were scanned, and their intensity was determined using ImageJ (NIH, Bethesda, MD, USA).

### 2.11. Molecular Docking

The 3D crystal structure of PPARγ was obtained from Protein Data Bank, (http://www.rcsb.org/pdb) (PDB, ID: 2PRG). Macromolecules were viewed and separated from ligand, solvent and unneeded residues by using UCSF Chimera [54]. Isolated protein receptor was prepared for docking by optimization by means of Autodock Tools (ADT) v1.5.6. This optimization comprises the addition of polar hydrogens and setting the grid box according to the best configuration of the active site amino acid residues [55]. The size of grid box was set at 30 x 36 x 26 (x,y,z). The center of the grid box was located at 59.388 × −6.279 × 36.212 (x,y,z). The 2D structure of the isolated compounds (**1**–**6**) was drawn using ChemBioDraw software, then converted to .pdb 3D structures using UCSF Chimera [54]. Ligands were optimized by using ADT v1.5.6. Molecular docking analysis for the isolated compounds (**1**–**6**) was carried out by AutoDock Vina (http://vina.scripps.edu/), and the obtained results were analyzed and visualized using PyMOL v2.3.2.

### 2.12. Statistical Analysis

The results were presented as mean ± SEM (standard error of mean). All statistical comparisons were made by one-way ANOVA followed by Tukey’s test and the differences were considered statistically significant at *P* < 0.05. The statistical analysis was carried out using GraphPad Prism 7 (La Jolla, CA, USA).

## 3. Results

### 3.1. Identification of the Isolated Compounds from PPE

**Figure 1:**
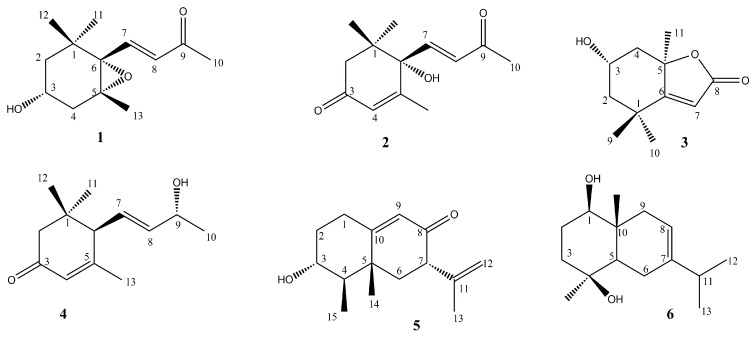
Chemical structure of the isolated compounds.

The structure elucidation of the isolated compounds was performed by comparing their spectral data (^1^H-NMR, ^13^C-NMR and MS) and physicochemical properties with those previously published. The isolated compounds (Figure 1) were found to be 3α-hydroxy-5,6-epoxy-7-megastigmen-9-one (**1**) [56], (+)-dehydrovomifoliol (**2**) [57], loliolide (**3**) [58], (6R,7E,9R)-9-hydroxy-4,7-megastigmadien-3-one (**4**) [59], petasol (**5**) [60] and oplodiol (**6**) [61].

### 3.2. PPE Ameliorates Hyperglycemia and IR in HFD/STZ-Induced Rats

**Figure 2:**
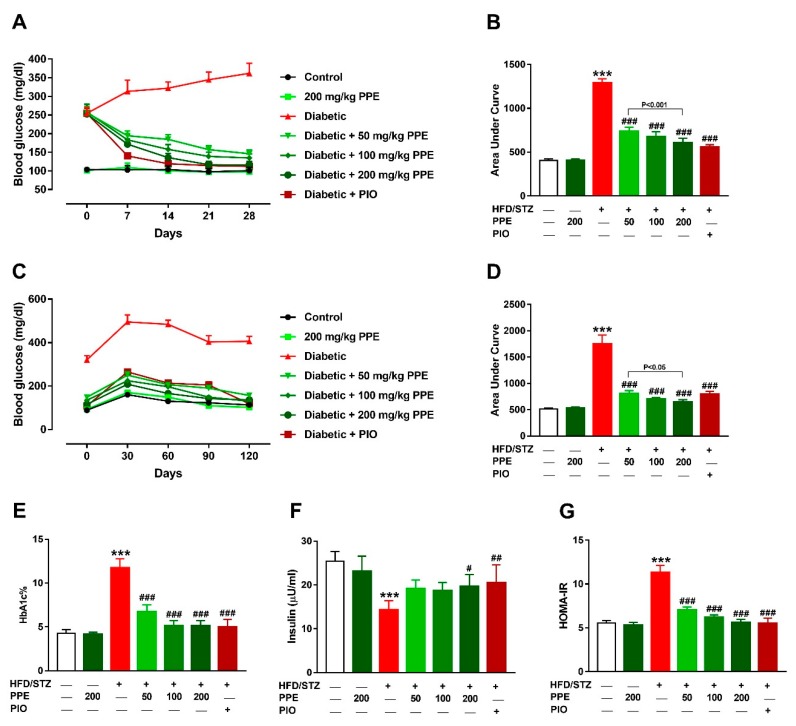
*P. pavonia* extract (PPE) ameliorates hyperglycemia and insulin resistance (IR) in high fat diet/streptozotocin (HFD/STZ)-induced rats. PPE and pioglitazone (PIO) ameliorated blood glucose levels (**A**,**B**), glucose tolerance (**C**,**D**), and HbA1c% (**E**), increased serum insulin (**F**) and decreased HOMA-IR (**G**) in STZ/HFD-induced rats. Data are mean ± SEM, *n* = 6. *** *P* < 0.001 versus control and ^#^ *P* < 0.05, ^##^ *P* < 0.01 and ^###^ *P* < 0.001 versus diabetic.

Diabetic rats showed a significant hyperglycemia throughout the experiment (Figure 2A). OGTT of the diabetic rats revealed significant increase in blood glucose levels at 0, 30, 60, 90, and 120 min (Figure 2C). Area under curve analysis of the blood glucose during the treatment period (Figure 2B) and OGTT (Figure 2D) revealed significant hyperglycemia in HFD/STZ-induced rats and the anti-hyperglycemic effect of PPE and PIO. PPE exerted a dose-dependent anti-hyperglycemic effect in diabetic rats, with no effect in normal rats. Hyperglycemia in HFD/STZ-induced rats was confirmed by the significantly elevated HBA1c% (*P* < 0.001), an effect that was remarkably attenuated by PPE and PIO (Figure 2E).

Serum insulin was decreased in HFD/STZ-induced rats (Figure 2F), and treatment with 50 and 100 mg/kg PPE failed to increase insulin significantly in diabetic rats, whereas the 200 mg/kg (*P* < 0.05) and PIO (*P* < 0.01) significantly improved serum insulin levels. Given that the assay of glucose or insulin can poorly detect impaired insulin sensitivity [62], IR was evaluated by determining HOMA-IR which was significantly increased in diabetic rats (*P* < 0.001; Figure 2G). Treatment with 50, 100, and 200 mg/kg PPE and PIO decreased HOMA-IR significantly.

### 3.3. PPE Improves Liver Glucose Metabolizing Enzymes and Glycogen in HFD/STZ-Induced Rats

**Figure 3:**
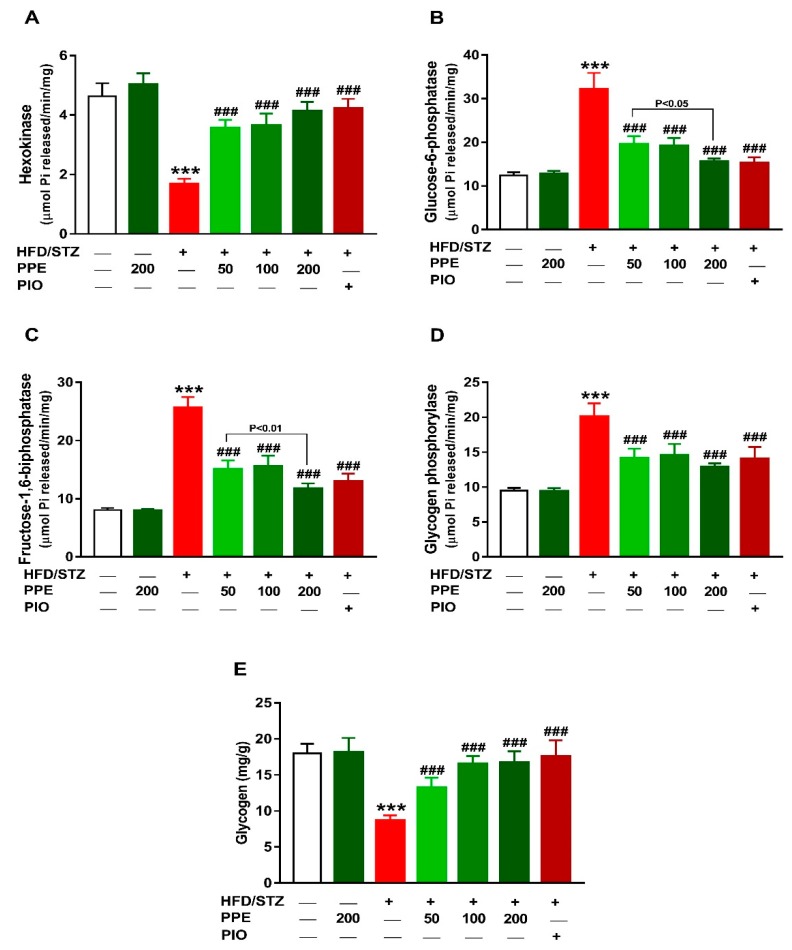
PPE improves liver glucose-metabolizing enzymes and glycogen in HFD/STZ-induced rats. PPE and PIO increased hexokinase activity (**A**), suppress glucose-6-phosphatase (**B**), fructose-1,6-biphosphatase (**C**) and glycogen phosphorylase (**D**), and improved glycogen content (**E**) in diabetic rats. Data are mean ± SEM, *n* = 6. *** *P* < 0.001 versus control and ^###^ *P* < 0.001 versus diabetic.

To explore the anti-hyperglycemic mechanism of PPE in diabetic rats, glucose-metabolizing enzymes and glycogen content were determined. Hexokinase activity was noticeably decreased in the liver of HFD/STZ rats (*P* < 0.001; Figure 3A). In contrast, G-6-Pase (Figure 3B), F-1,6-BPase (Figure 3C), and glycogen phosphorylase (Figure 3D) were increased in the diabetic rats. Liver glycogen content was diminished in the diabetic group when compared with the control rats (*P* < 0.001; Figure 3E). Oral supplementation of PPE and PIO increased hepatic hexokinase activity and glycogen, and suppressed G-6-Pase, F-1,6-BPase, and glycogen phosphorylase HFD/STZ-induced rats. Of note, PPE decreased the activity of G-6-Pase and F-1,6-BPase dose-dependently in diabetic rats, whereas exerted no effect in normal rats.

### 3.4. PPE Attenuates Hyperlipidemia in HFD/STZ-Induced Rats

**Figure 4:**
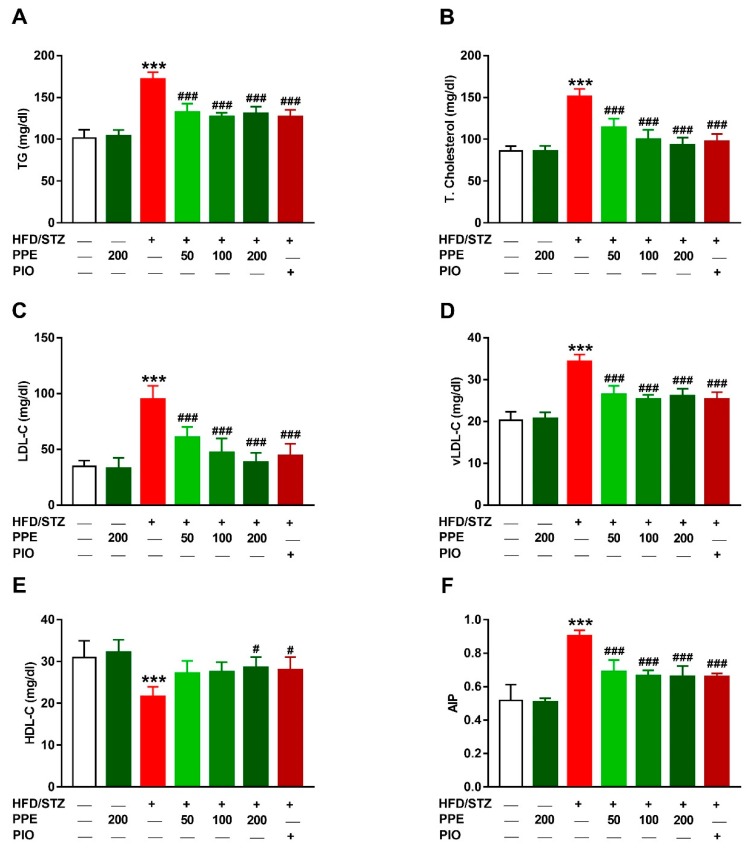
PPE attenuates hyperlipidemia in HFD/STZ-induced rats. PPE and PIO decreased serum triglycerides (**A**), total cholesterol (**B**), LDL-C (**C**), vLDL-C (**D**) and atherogenic index of plasma (**F**), and increased HDL-C (**E**) in diabetic rats. Data are mean ± SEM, *n* = 6. *** *P* < 0.001 versus control and ^#^ *P* < 0.05 and ^###^ *P* < 0.001 versus diabetic.

To evaluate the anti-hyperlipidemic effect of PPE in diabetic rats, serum lipids were determined. HFD/STZ-induced rats exhibited significant hypertriglyceridemia (Figure 4A) and hypercholesterolemia (Figure 4B) when compared with the control group (*P* < 0.001). LDL-C (Figure 4C), vLDL-C (Figure 4D) and AIP (Figure 4F) were increased in HFD/STZ-induced rats, whereas HDL-C was significantly declined (Figure 4E). Treatment of the diabetic rats with PPE (50, 100, and 200 mg/kg) as well as PIO reduced serum TG, TG, LDL-C, vLDL-C, and AIP. The 200 mg/kg dose of PPE increased HDL-C levels in diabetic rats and exerted no effect on lipid profile in normal rats.

### 3.5. PPE Prevents Liver Injury and Lipid Accumulation in HFD/STZ-Induced Rats

**Figure 5:**
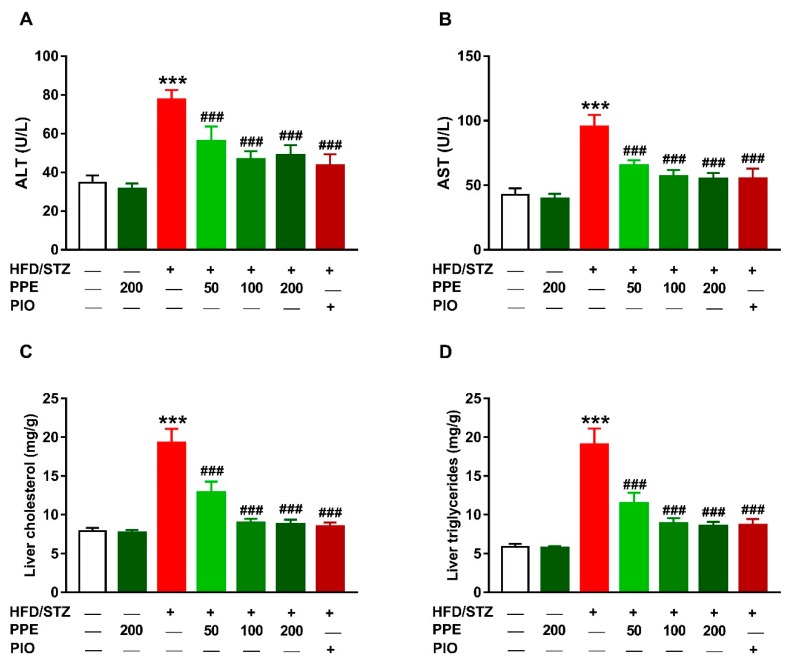
PPE and PIO decreased serum ALT (**A**) and alanine aminotransferase (AST) (**B**), and liver cholesterol (**C**) and triglycerides (**D**) in diabetic rats. Data are mean ± SEM, *n* = 6. *** *P* < 0.001 versus Control and ^###^ *P* < 0.001 versus diabetic.

**Figure 6:**
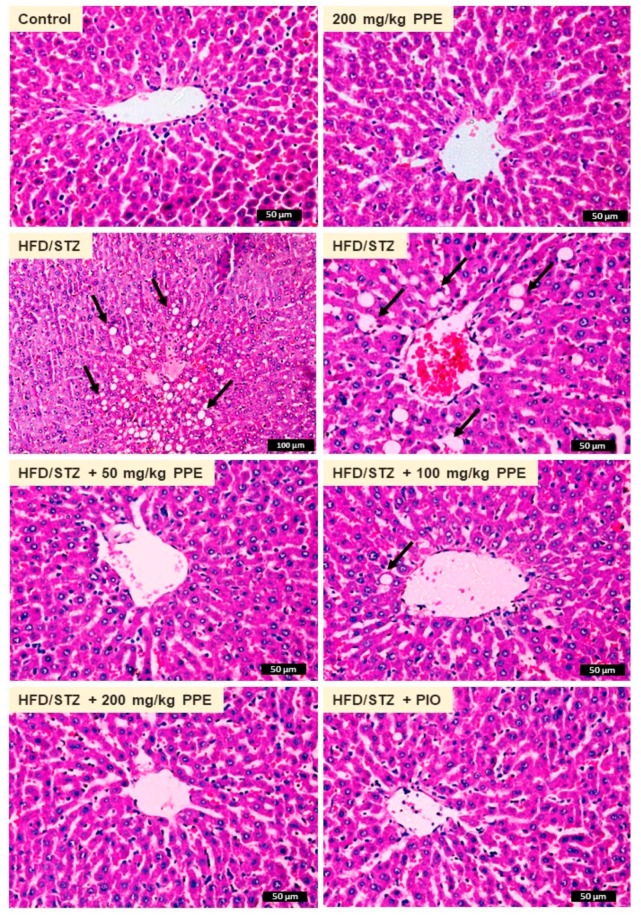
Photomicrographs of H&E-stained liver sections in liver of control and PPE-supplemented rats showing normal structure of hepatic lobule, hepatocytes, and central vein. HFD/STZ-induced rats showed centrilobular hepatic vacuolation of round border and clear vacuoles consistent with fatty changes (arrows). Diabetic rats treated with 50, 100, and 200 mg/kg PPE or PIO showed remarkable improvement of liver structure.

The protective effect of PPE on diabetes-induced liver dysfunction was evaluated through the assessment of serum transaminases, liver cholesterol and TG, and histopathological changes. HFD/STZ-induced rats exhibited significantly elevated serum ALT (Figure 5A) and AST (Figure 5B), and hepatic cholesterol (Figure 5C) and TG (Figure 5D). These biochemical findings were confirmed by the histological investigation where the HFD/STZ-induced rats showed centrilobular hepatic vacuolation of round border and clear vacuoles consistent with fatty changes (Figure 6).

Oral administration of PPE (50, 100, and 200 mg/kg) and PIO reduced serum ALT and AST, and liver cholesterol and TG (Figure 5) and prevented hepatic steatosis (Figure 6) in diabetic rats. Rats received 200 mg/kg PPE for 4 weeks showed normal liver function (Figure 5) and histological architecture (Figure 6).

### 3.6. PPE Attenuates Oxidative Stress and Enhances Antioxidants in HFD/STZ-Induced Rats

**Figure 7:**
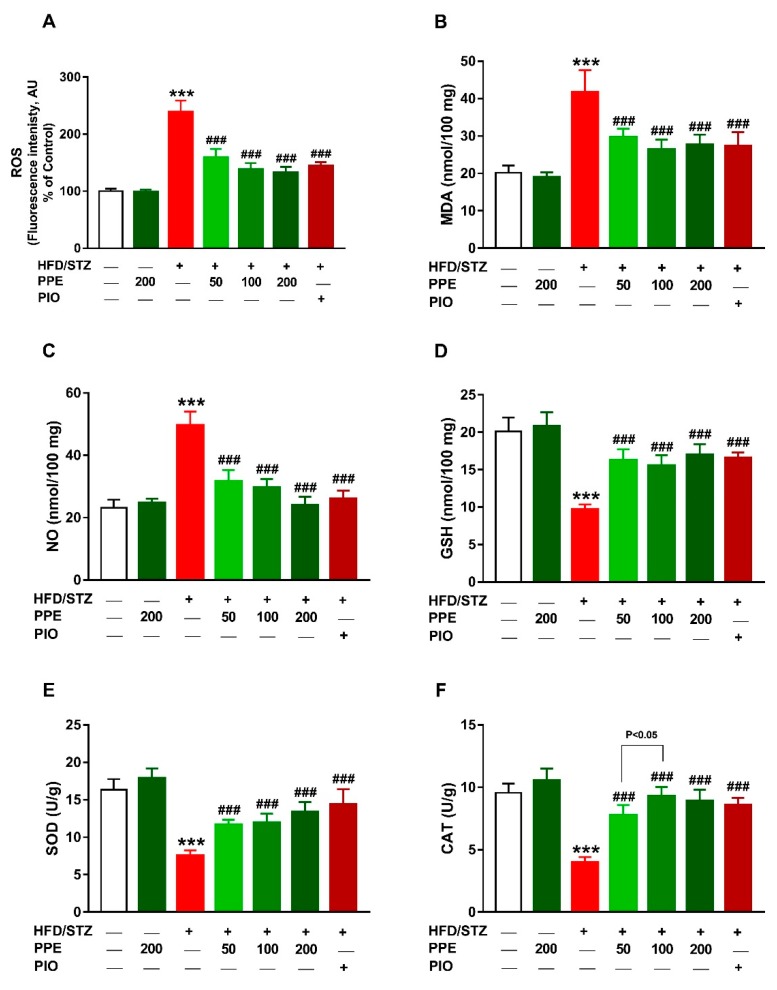
PPE attenuates oxidative stress and enhances antioxidants in HFD/STZ-induced rats. PPE and PIO decreased ROS (**A**), MDA (**B**), and NO (**C**), and increased GSH (**D**), SOD (**E**), and CAT (**F**) in liver of diabetic rats. Data are mean ± SEM, *n* = 6. *** *P* < 0.001 versus control and ^###^ *P* < 0.001 versus diabetic.

The protective effect of PPE on oxidative stress in diabetic animals was assessed through the assessment of hepatic ROS, LPO, and NO, and antioxidant defenses (Figure 7). Rats received HFD and STZ showed hepatic oxidative stress manifested by the significantly increased ROS, LPO, and NO when compared with the control animals (*P* < 0.001; Figure 7A–C). Additionally, hepatic GSH (Figure 7D), SOD (Figure 7E), and CAT (Figure 7F) were markedly reduced in HFD/STZ-induced rats (*P* < 0.001). Oral administration of PPE or PIO suppressed ROS, LPO, and NO and enhanced hepatic GSH, SOD, and CAT (Figure 7). Normal rats received 200 mg/kg PPE for 4 weeks showed no changes in hepatic ROS, LPO, NO, and antioxidants.

### 3.7. PPE Suppresses Inflammation in HFD/STZ-Induced Rats

**Figure 8:**
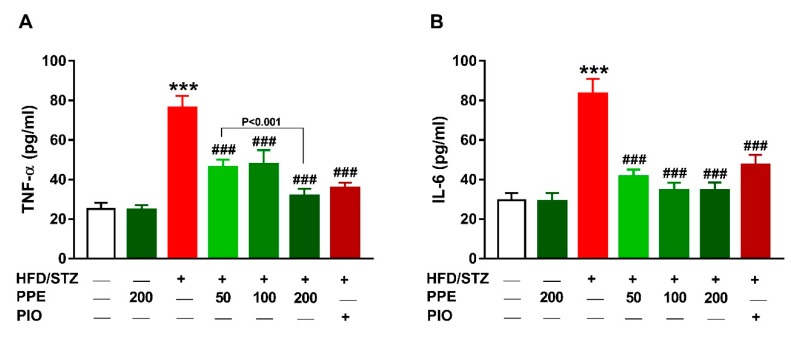
PPE and PIO decreased serum TNF-α (**A**) and IL-6 (**B**) in HFD/STZ-induced rats. Data are mean ± SEM, *n* = 6. *** *P* < 0.001 versus control and ^###^ *P* < 0.001 versus diabetic.

The anti-inflammatory effect of PPE in diabetic rats was evaluated by determining serum TNF-α (Figure 8A) and IL-6 (Figure 8B). The circulating levels of TNF-α and IL-6 were increased significantly in diabetic rats (*P* < 0.001) and treatment with PPE or PIO ameliorated these cytokines. Oral supplementation of 200 mg/kg PPE did not alter serum TNF-α and IL-6 in normal rats.

### 3.8. PPE Upregulates Hepatic PPARγ in HFD/STZ-Induced Rats

**Figure 9:**
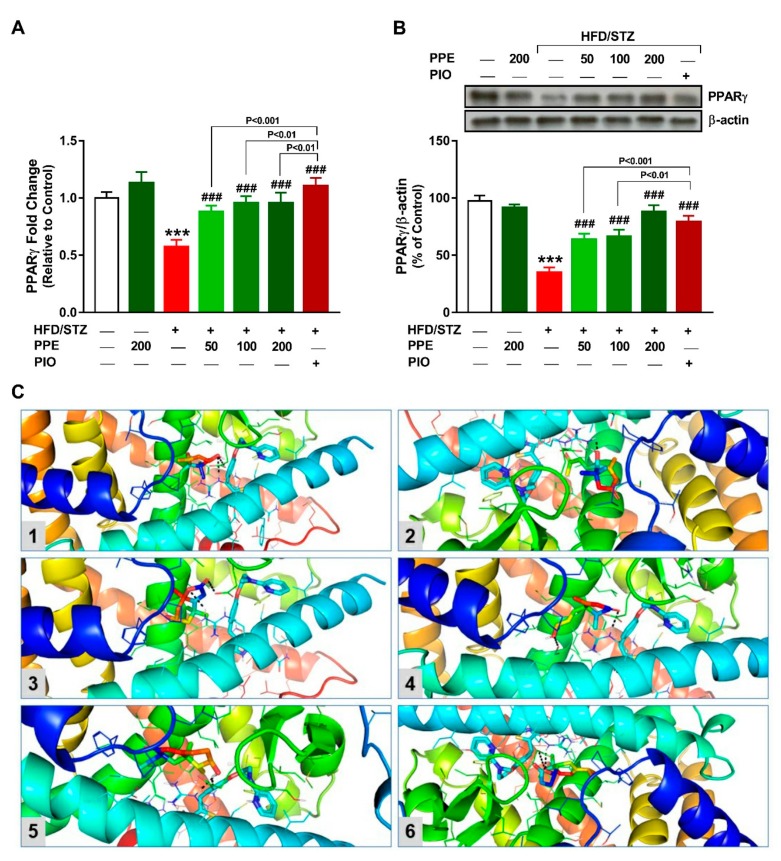
PPE upregulates hepatic PPARγ in HFD/STZ-induced rats. PPE and PIO increased mRNA abundance (**A**) and protein expression (**B**) levels of PPARγ in the liver of diabetic rats. Data are mean ± SEM, *n* = 6. *** *P* < 0.001 versus control and ^###^ *P* < 0.001 versus diabetic. (**C**) Molecular docking analysis showing the binding between *P. pavonia*-derived terpenes (Compounds **1**–**6**) and PPARγ. Compounds **1**, **5,** and **6** interact with Arg288 and Glu343, compounds **2** and **3** interact with Arg288 and compound **4** binds Arg288 and Glu295 in PPARγ.

To investigate the role of PPARγ in mediating the beneficial effects of PPE in diabetic rats, we determined its gene and protein expression, and conducted a molecular docking study to assess its binding with the isolated compounds. There was a significant downregulation of hepatic PPARγ mRNA (Figure 9A) and protein expression (Figure 9B) in HFD/STZ diabetic rats. PPE and PIO administration significantly increased PPARγ mRNA and protein expression in diabetic rats.

The binding affinity of the isolated compounds (**1**–**6**) toward PPARγ was studied using molecular docking. The compounds showed binding in the range of −6.2 (kcal/mol) to −7.4 (kcal/mol) (Table 3). Compound **5** showed the lowest binding energy toward PPARγ, representing the most stable complex (Figure 9C). Docking analysis of the compounds **1**, **5,** and **6** revealed binding affinities toward Arg288 and Glu343, whereas compounds **2** and **3** showed affinities toward Arg288 and compound **4** binds Arg288 and Glu295 (Figure 9C).

## 4. Discussion

T2DM is characterized by IR accompanied with insufficient insulin secretion, resulting in hyperglycemia that can promote organ damage and serious complications [3]. Therefore, management of IR and hyperglycemia is critical for the treatment of DM and prevention of its complications. Herein, we investigated the potential of a terpenoid rich fraction of the marine alga *P. pavonia* to ameliorate hyperglycemia, IR, hyperlipidemia, and oxidative stress in HFD/STZ-induced diabetic rats, pointing to the possible involvement of PPARγ.

HFD/STZ-induced diabetes has been a widely accepted model that mimic human T2DM. Feeding a HFD to rodents induces IR and metabolic derangements [63], and STZ was evidenced to damage pancreatic β-cells resulting in decreased insulin release [64]. Therefore, the T2DM model induced by HFD and STZ is used frequently to understand the pathogenesis of DM and to evaluate the beneficial effects of new therapeutic agents. Accordingly, HFD/STZ-induced rats in this study exhibited hyperglycemia, and impaired glucose tolerance and insulin secretion. In addition, HbA1c was significantly elevated in HFD/STZ-induced rats, demonstrating the development of hyperglycemia and diabetes. HbA1c is a valuable indicator for the diagnosis and management of hyperglycemia in diabetes and levels lower than 7% indicate good glycemic control [65]. Previous work from our lab [15] as well as others [66,67] have shown hyperglycemia, elevated HbA1c% and altered insulin secretion, and sensitivity in HFD/STZ-induced rodents. In addition, the values of HOMA-IR suggested the development of IR in HFD/STZ-induced rats. Interestingly, treatment of the diabetic rats with different doses of PPE ameliorated hyperglycemia, glucose intolerance, and HbA1c%, indicating its potent anti-hyperglycemic efficacy. PPE increased insulin release and improved insulin sensitivity as demonstrated by the significant reduction of HOMA-IR. The anti-diabetic effect of *P. pavonia* hydroethanolic extract has been previously reported in STZ/nicotinamide (NA) diabetic rats [68]. We have reported significant improvement of glucose tolerance and insulin sensitivity following treatment of the STZ/NA-induced rats with 100 mg/kg *P. pavonia* hydroethanolic extract [68]. Here, we introduced new information that a terpenoid rich fraction of *P. pavonia* can effectively ameliorate hyperglycemia and IR in type 2 diabetic rats.

Liver plays a key role in glucose homeostasis and uncontrolled hepatic gluconeogenesis and glycogenolysis are centrally implicated in hyperglycemia [69]. In addition, impaired insulin release and/or action causes hyperglycemia through increasing hepatic glucose output and decreasing glucose utilization by peripheral tissues [69]. The enzymes regulating hepatic glucose production are potential targets for the control of glucose homeostasis in diabetes. Therefore, we determined the effect of PPE on hepatic glucose-metabolizing enzymes and glycogen in diabetic rats. Hexokinase activity was decreased whereas G-6-Pase and F-1,6-BPase were significantly increased in the liver of HFD/STZ-induced rats as previously reported [70,71]. Hexokinase is an insulin-dependent enzyme involved in the oxidation of glucose and its activity is decreased in insulin deficiency and IR [72,73]. The declined hepatic hexokinase activity in diabetes results in decreased glucose oxidation rats via glycolysis, leading to hyperglycemia as shown in the current study. In contrast, G-6-Pase and F-1,6-BPase were increased in diabetic rats. Both enzymes are involved in the regulation of glucose homeostasis through eliciting gluconeogenesis. F-1,6-BPase catalyzes the dephosphorylation of fructose-1,6-bisphosphate to fructose-6-phosphate [74] and G-6-Pase completes the ending step in gluconeogenesis and glycogenolysis by catalyzing the dephosphorylation of glucose-6-phosphate to glucose [75]. In diabetes, the increase in hepatic glucose production is directly associated with impaired suppression of G-6-Pase [76]. The increased activity of both G-6-Pase and F-1,6-BPase is directly attributed to impaired insulin release. Accordingly, hexokinase was diminished and G-6-Pase and F-1,6-BPase were activated in the liver of diabetic rats exhibiting insulin insufficiency [70,71,73]. Consequently, hepatic glycogen phosphorylase activity was increased, and glycogen content was declined in HFD/STZ diabetic rats, demonstrating exaggerated glycogenolysis and gluconeogenesis. Glycogen is the storage form of glucose in liver and skeletal muscle and plays an important role in glucose homeostasis. Given that insulin stimulates glycogenesis by stimulating glycogen synthase, and suppressing glycogen phosphorylase and G-6-Pase, decreased glycogen levels reflect insulin insufficiency and/or resistance [77,78]. PPE significantly improved hexokinase activity and suppressed F-1,6-BPase, G-6-Pase, and glycogen phosphorylase, resulting in restoration of the hepatic glycogen content. Hence, it is noteworthy assuming that activation of hexokinase secondary to increased insulin secretion and sensitivity enhanced glucose oxidation and glycogenesis in the liver of HFD/STZ-induced rats. In addition, amelioration of fasting blood glucose in diabetic rats treated with PPE is a direct result of increased hexokinase activity and consequently glucose oxidation.

Dyslipidemia is one of the most common complications seen in diabetes irrespective of insulin deficiency or IR [7]. Diabetic dyslipidemia is featured with increased serum TG, TC, and LDL-C and decreased HDL-C, with hypertriglyceridemia representing the most common abnormality [79]. HFD/STZ diabetic rats in the present investigation exhibited atherogenic lipid profile manifested by increased TG, TC, LDL-C, and vLDL-C, and decreased HDL-C as previously demonstrated [80]. Administration of PPE significantly decreased serum TG, TC, LDL-C, vLDL-C, and AIP, a frequent predictor of atherosclerosis, and the high dose markedly increased serum HDL-C. Given the role of insulin in promoting the storage of lipids, the anti-hyperlipidemic effect of PPE could be mediated, at least in part, via increasing insulin sensitivity and PPARγ activation. This notion is supported by the improvement of lipid profile in PIO-treated diabetic rats. PIO is PPARγ agonist that ameliorates IR and improves TG by enhancing vLDL-TG removal from the circulation and increased HDL-C levels [81,82].

Diabetes can accelerate the progression of liver injury, thereby aggravating abnormal glucose and lipid metabolism [4]. In addition, the accumulation of lipids in the liver may worsen IR, leading to severe metabolic alterations. Excessive accumulation of hepatic lipids and hyperglycemia can provoke hepatocyte injury and liver dysfunction [83]. Here, HFD/STZ-induced diabetic rats showed liver injury evidenced by the elevated serum ALT and AST, the indicators of liver function. Increased serum transaminases has been associated with hepatic IR [84] and rats received HFD and/or STZ showed elevated serum ALT and AST [14]. In addition, hepatic TG and TC were increased in HFD/STZ-induced rats in the present investigation. These biochemical findings were supported by the histological examination which showed centrilobular hepatic vacuolation of round border and clear vacuoles consistent with fatty changes. Hepatic lipid infiltration occurs as a consequence to IR-related lipolysis which promote dyslipidemia and increase fatty acids taken up by the liver as an energy source. Accumulated fatty acids increases lipogenesis and disturbs mitochondrial β-oxidation, resulting in further lipid infiltration in the liver [85].

Hyperglycemia and hyperlipidemia were associated with increased generation of ROS, oxidative stress, and inflammation [15,86]. The role of ROS in liver injury has been established by studies showing that reduction of ROS can protect against hepatocyte damage induced by several insults [87,88,89]. HFD/STZ diabetic rats in this study showed excessive production of ROS associated with increased MDA and NO. Increased ROS can promote oxidative damage to lipids, proteins, and DNA [90,91]. Although the liver is equipped with antioxidant defenses, including GSH, SOD, and CAT, hyperglycemia-induced ROS generation was associated with diminished levels of these antioxidants as previously demonstrated [14,15]. Excess ROS can also activate NF-κB, a transcription factor eliciting the expression of TNF-α, IL-6, and inducible NO synthase (iNOS). These ROS-mediated effects explain the elevated hepatic MDA and NO levels as well as the pro-inflammatory cytokines in diabetic rats. In this context, circulating levels of TNF-α and IL-6, and liver NF-κB expression have been reported to increase in HFD/STZ-induced rats [14,15]. In patients with T2DM, increased circulating levels of TNF-α and IL-6 was reported [13]. Increased IL-6 is associated with impaired glucose tolerance [16] as well as IR in hepatocytes [17]. IL-6 reduces insulin receptor substrate (IRS)-1 tyrosine phosphorylation and inhibits insulin-dependent activation of protein kinase B/Akt in hepatocytes [17]. TNF-α impairs the ability of insulin to stimulate peripheral glucose uptake and to suppress hepatic glucose production [18], increases lipolysis in adipocytes [19], and impairs insulin signaling in muscle cells [20]. Therefore, counteracting oxidative stress and inflammation can result in improved insulin sensitivity.

PPE attenuated diabetes-associated liver injury and lipid accumulation in HFD/STZ-induced rats. All doses of PPE reduced serum transaminases and hepatic TC and TG levels and prevented liver injury in HFD/STZ-induced rats. In addition, PPE suppressed ROS generation, LPO and pro-inflammatory cytokines and boosted GSH and antioxidant enzymes in the liver of HFD/STZ-induced rats. Attenuation of ROS and inflammation significantly contributed to the improvement of insulin sensitivity and suppression of hepatic steatosis. We have previously demonstrated the hepatoprotective efficacy of *P. pavonia* ethanolic extract in azoxymethane-induced mice [32]. *P. pavonia* ameliorated liver function, prevented histological alterations, attenuated oxidative stress and NF-κB and increased the expression of PPARγ in liver [32] and colon [31] of azoxymethane-induced mice. Therefore, we thought that PPARγ mediated, at least in part, the beneficial effects of *P. pavonia*-derived terpenes in diabetic rats. To explore the possible involvement of PPARγ, we determined the effect of PPE on its gene and protein expression and conduced a molecular docking to study its binding with the isolated terpenes. Interestingly, PPE increased both mRNA abundance and protein expression levels of hepatic PPARγ in diabetic rats. PPARγ agonists such as PIO function as insulin sensitizers that enhance insulin action, ameliorate hyperglycemia in T2DM, lower plasma free fatty acids and TG, modulate the expression of inflammatory cytokines and prevent excessive lipid accumulation in liver and other peripheral tissues [22]. Hence, it is noteworthy assuming that the antihyperglycemic, insulin-sensitizing and anti-hyperlipidemic effects of PPE were mediated via PPARγ activation. The ability of PPE to activate PPARγ was supported by the molecular docking which revealed that compounds **1**, **3**, **5**, and **6** possess binding affinity toward Arg288 and Glu343, compound **2** showed binding affinity toward Arg288 and compound **4** bound to Arg288 and Glu295. In addition to the metabolic effects, PPARγ activation is associated with suppressed inflammation and increased expression of antioxidant enzymes. PPARγ prevents NF-κB-dependent inflammatory genes transcription by inhibiting IκBα degradation, suppressing p65 nuclear translocation [92], and reducing NF-κB-dependent transcriptional control [93]. Moreover, activation of PPARγ can directly promote the expression of antioxidant enzymes [94] and suppress NADPH oxidase-dependent ROS production [95]. Besides binding PPARγ, *P. pavonia*-derived terpenes may exert free radical-scavenging and anti-inflammatory activities. However, studies scrutinizing the antioxidant and anti-inflammatory activities of the isolated terpenes are scarce. For instance, loliolide has been recently reported to suppress oxidative stress and increase the expression of heme oxygenase-1 in H_2_O_2_-induced HaCaT cells [96].

## 5. Conclusions

**Figure 10:**
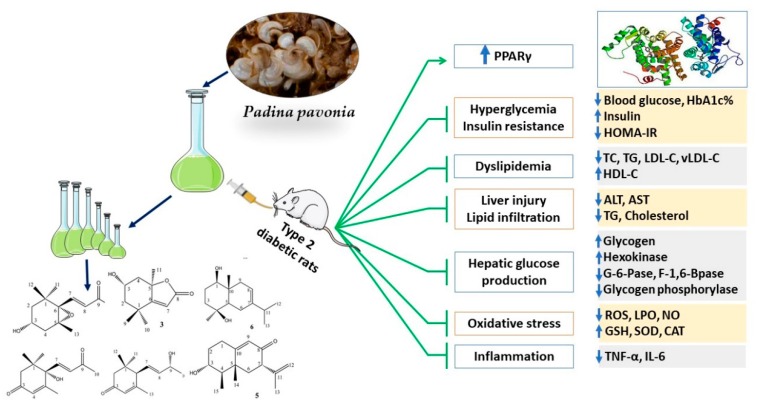
A schematic diagram showing the antidiabetic properties of *P. pavonia* terpenoids-rich extract.

This study confers new information on the antidiabetic potential of *P. pavonia*-derived terpenes. Treatment of T2DM rats with PPE ameliorated hyperglycemia, glucose intolerance, and IR. PPE increased hepatic hexokinase activity and glycogen, and suppressed G-6-Pase, F-1,6-BPase, and glycogen phosphorylase, suggesting its efficacy to improve carbohydrate metabolism and reduce hepatic glucose production. In addition, PPE prevented hyperlipidemia and inflammation, and protected the liver against injury, lipid accumulation, and oxidative stress. These beneficial effects of PPE are associated with PPARγ activation and in silico investigations showed the binding affinity of the isolated terpenes toward PPARγ. The antidiabetic effects of *P. pavonia*-derived terpenes are summarized in Figure 10. Therefore, *P. pavonia*-derived terpenes represent promising leads in the development of effective anti-diabetic agents, pending further studies to explore the underlying mechanisms.

## Figures and Tables

**Table 1:** NMR data of compounds **1**, **2,** and **3**.

	1		2		3	
Position	δ_H_ (*J* in Hz)	δ_C_	δ_H_ (*J* in Hz)	δ_C_	δ_H_ (*J* in Hz)	δ_C_
1		37.58		42.51		35.38
2	1.66 (1H, *m*, H-2α) 1.33 (1H, *m*, H-2β)	49.21	2.64 (1H, *d*, *J =* 16.7, H-2α), 2.41 (1H, *d*, *J =* 16.7, H-2β)	48.95	1.94 (1H, *dd*, *J* = 13.4; 2.7, H-2α), 1.33 (1H, d*t*, *J* = 13.11, 3.4, H-2β)	50.14
3	3.89 (1H, *m*)	63.72		197.87	4.01 (1H, *m*, H-3)	63.75
4	1.71 (1H, *m*, H-4α), 2.43 (1H, *dd*, *J* = 13.9, 4.8, H-4β)	41.49	6.12 (1H, br. *s*)	129.11	2.87 (1H, *dd*, *J* = 13.4; 2.7, H-4α), 1.22 (1H, d*t*, *J* = 12.67, 3.6, H-4β)	48.87
5		68.53		162.31		88.33
6		70.16		80.19		184.93
7	δ 7.15 (1H, *d*, *J =* 14.8)	143.57	6.91 (1H, *d*, *J =* 15.2)	147.14	5.91 (1H, *s*, H-7)	116.51
8	6.34 (1H, *d*, *J =* 14.8)	133.65	6.52 (1H, *d*, *J =* 15.2)	133.16		173.64
9		196.44		197.15	1.28 (3H, *s*, H-9)	25.26
10	2.32 (3H, *s*, H-10)	27.76	2.29 (3H, *s*)	30.01	1.17 (3H, *s*, H-10)	30.29
11	0.97 (3H, *s*, H-11)	26.25	1.22 (3H, *s*)	22.94	1.62 (3H, *s*, H-11)	26.62
12	1.19 (3H, *s*)	28.92	1.11 (3H, *s*)	25.49		
13	1.19 (3H, *s*)	20.87	1.93 (3H, *d*, *J =* 1.2)	19.95		
14						
15						
3-OH					4.92 (1H, s,3-OH)	

**Table 2:** NMR data of compounds **4**, **5** and **6**.

	4		5		6	
Position	δ_H_ (*J* in Hz)	δ_C_	δ_H_ (*J* in Hz)	δ_C_	δ_H_ (*J* in Hz)	δ_C_
1		36.25	2.67 (1H, t*dd*, *J* = 17.3, 6.1, 2.4), 2.52 (1H, *ddd*, J = 17.3, 5.9, 3.1)	29.75	3.74 (1H, *dd*, *J* = 12.5, 4.5)	80.14
2	2.54 (1H, *d*, *J* = 15.9, H-2α), 2.12 (1H, *d*, *J* = 15.9, H-2 β)	47.14	2.29 (1H, *m*) 1.51 (1H, *m*	33.11	1.91–1.98 (2H, *m*, H-2, H-9), 1.83 (1H, d*t*, *J* = 14.4, 4)	28.18
3		198.9.72	3.75 (1H, t*d*, *J* = 12.1, 5.2)	69.63	1.61–1.74 (2H, *m*)	41.59
4	5.93 (1H, *s*)	125.98	1.42 (1H, *m*)	49.81		71.64
5		164.89		37.96	1.46 (1H, *dd*, *J* = 12.2, 6.1)	48.75
6	2.87 (1H, *d*, *J* = 8.7)	54.93	2.11 (1H, *dd*, *J* = 14.3, 5.8), 1.91 (1H, *t*, *J* = 14.8)	39.52	2.05–2.18 (3H, *m*, H-6β, H-9)	25.09
7	5.62 (1H, *dd*, *J* = 14.3, 8.7)	126.36	3.54 (1H, *dd*, J = 15.2, 5.7)	49.78		143.67
8	5.71 (1H, *dd*, *J* = 14.3, 5.7)	139.19		197.84	5.65 (1H, *d*, *J* = 5.1)	118.32
9	4.45 (1H, *dq*, *J* = 5.7, 6.2)	65.87	5.84 (1H, *d*, *J* = 2.3)	122.75	2.05–2.18 (3H, *m*, H-6β, H-9), 1.91–1.98 (2H, *m*, H-2, H-9)	43.81
10	1.32 (3H, *d*, *J* = 6.2)	22.91		164.98		39.15
11	1.05 (3H, *s*)	26.78		141.57	2.44 (1 H, *septet*, *J* = 7.4)	36.32
12	1.14 (3H, *s*)	27.36	5.12 (1H, *br*, *t*, *J* = 1.8) 4.91 (1H, *br*, *s*)	110.56	1.10 (3H, *d*, *J* = 7.5)	22.98
13	1.98 (3H, *s*)	22.42	1.83 (3H, *br*, *s*)	19.64	1.12 (3H, *d*, *J* = 7.5)	22.75
14			1.26 (3H, *s)*	15.38	1.02 (3H, *s*)	13.53
15			1.17 (3H, *d*, *J* = 7.4)	10.32	1.23 (3H, *s*)	30.43

**Table 3:** Binding affinities of isolated compounds (**1**–**6**) with PPARγ.

Compound	Affinity (kcal/mol)	Polar Bonds
**1**	−7.0	2
**2**	−6.2	1
**3**	−6.7	1
**4**	−7.1	2
**5**	−7.4	2
**6**	−7.0	2

## References

[1] Cho N.H., Shaw J.E., Karuranga S., Huang Y., Da Rocha Fernandes J.D., Ohlrogge A.W., Malanda B. (2018). Idf diabetes atlas: Global estimates of diabetes prevalence for 2017 and projections for 2045. Diabetes Res. Clin. Pract..

[2] Kahn S.E., Cooper M.E., Del Prato S. (2014). Pathophysiology and treatment of type 2 diabetes: Perspectives on the past, present, and future. Lancet.

[3] Jellinger P.S. (2007). Metabolic consequences of hyperglycemia and insulin resistance. Clin. Cornerstone.

[4] Armstrong M.J., Adams L.A., Canbay A., Syn W.K. (2014). Extrahepatic complications of nonalcoholic fatty liver disease. Hepatology.

[5] Petersen M.C., Shulman G.I. (2018). Mechanisms of insulin action and insulin resistance. Physiol. Rev..

[6] Halban P.A., Polonsky K.S., Bowden D.W., Hawkins M.A., Ling C., Mather K.J., Powers A.C., Rhodes C.J., Sussel L., Weir G.C. (2014). Beta-cell failure in type 2 diabetes: Postulated mechanisms and prospects for prevention and treatment. Diabetes Care.

[7] Taskinen M.R. (2003). Diabetic dyslipidaemia: From basic research to clinical practice. Diabetologia.

[8] Samuel V.T., Shulman G.I. (2012). Mechanisms for insulin resistance: Common threads and missing links. Cell.

[9] Newsholme P., Cruzat V.F., Keane K.N., Carlessi R., De Bittencourt P.I. (2016). Molecular mechanisms of ros production and oxidative stress in diabetes. Biochem. J..

[10] Tiwari B.K., Pandey K.B., Abidi A.B., Rizvi S.I. (2013). Markers of oxidative stress during diabetes mellitus. J. Biomark..

[11] Bandeira Sde M., Guedes Gda S., Da Fonseca L.J., Pires A.S., Gelain D.P., Moreira J.C., Rabelo L.A., Vasconcelos S.M., Goulart M.O. (2012). Characterization of blood oxidative stress in type 2 diabetes mellitus patients: Increase in lipid peroxidation and sod activity. Oxidative Med. Cell. Longev..

[12] Rizvi S.I., Zaid M.A., Anis R., Mishra N. (2005). Protective role of tea catechins against oxidation-induced damage of type 2 diabetic erythrocytes. Clin. Exp. Pharmcol. Physiol..

[13] Pickup J.C., Chusney G.D., Thomas S.M., Burt D. (2000). Plasma interleukin-6, tumour necrosis factor α and blood cytokine production in type 2 diabetes. Life Sci..

[14] Sahin N., Orhan C., Erten F., Tuzcu M., Defo Deeh P.B., Ozercan I.H., Juturu V., Kazim S. (2019). Effects of allyl isothiocyanate on insulin resistance, oxidative stress status, and transcription factors in high-fat diet/streptozotocin-induced type 2 diabetes mellitus in rats. J. Biochem. Mol. Toxicol..

[15] Mahmoud A.M., Ashour M.B., Abdel-Moneim A., Ahmed O.M. (2012). Hesperidin and naringin attenuate hyperglycemia-mediated oxidative stress and proinflammatory cytokine production in high fat fed/streptozotocin-induced type 2 diabetic rats. J. Diabetes Complicat..

[16] Müller S., Martin S., Koenig W., Hanifi-Moghaddam P., Rathmann W., Haastert B., Giani G., Illig T., Thorand B., Kolb H. (2002). Impaired glucose tolerance is associated with increased serum concentrations of interleukin 6 and co-regulated acute-phase proteins but not tnf-alpha or its receptors. Diabetologia.

[17] Senn J.J., Klover P.J., Nowak I.A., Mooney R.A. (2002). Interleukin-6 induces cellular insulin resistance in hepatocytes. Diabetes.

[18] Lang C.H., Dobrescu C., Bagby G.J. (1992). Tumor necrosis factor impairs insulin action on peripheral glucose disposal and hepatic glucose output. Endocrinology.

[19] Green A., Dobias S.B., Walters D.J.A., Brasier A.R. (1994). Tumor necrosis factor increases the rate of lipolysis in primary cultures of adipocytes without altering levels of hormone-sensitive lipase. Endocrinology.

[20] Del Aguila L.F., Claffey K.P., Kirwan J.P. (1999). Tnf-α impairs insulin signaling and insulin stimulation of glucose uptake in C_2_C_12_ muscle cells. Am. J. Physiol. Endocrinol. Metab..

[21] Han L., Shen W.-J., Bittner S., Kraemer F.B., Azhar S. (2017). PPARs: Regulators of metabolism and as therapeutic targets in cardiovascular disease. Part ii: PPAR-β/δ and PPAR-γ. Future Cardiol..

[22] Tontonoz P., Spiegelman B.M. (2008). Fat and beyond: The diverse biology of ppargamma. Annu. Rev. Biochem..

[23] Zhang Q., Liu T., Ng C.Y., Li G. (2014). Diabetes mellitus and atrial remodeling: Mechanisms and potential upstream therapies. Cardiovasc. Ther..

[24] Mahmoud A.M., Hozayen W.G., Hasan I.H., Shaban E., Bin-Jumah M. (2019). Umbelliferone ameliorates CCl_4_-induced liver fibrosis in rats by upregulating ppargamma and attenuating oxidative stress, inflammation, and tgf-beta1/smad3 signaling. Inflammation.

[25] Mahmoud A.M., Mohammed H.M., Khadrawy S.M., Galaly S.R. (2017). Hesperidin protects against chemically induced hepatocarcinogenesis via modulation of nrf2/are/ho-1, ppargamma and tgf-beta1/smad3 signaling, and amelioration of oxidative stress and inflammation. Chem. Biol. Interact..

[26] Mahmoud A.M., Hussein O.E., Hozayen W.G., Abd El-Twab S.M. (2017). Methotrexate hepatotoxicity is associated with oxidative stress, and down-regulation of ppargamma and nrf2: Protective effect of 18beta-glycyrrhetinic acid. Chem. Biol. Interact..

[27] Kang H., Seo C., Park Y. (2015). Marine peptides and their anti-infective activities. Mar. Drugs.

[28] Kamenarska Z., Gasic M.J., Zlatovic M., Rasovic A., Sladic D., Kljajic Z., Stefanov K., Seizova K., Najdenski H., Kujumgiev A. (2002). Chemical composition of the brown alga *Padina pavonia* (l.) gaill. From the adriatic sea. Bot. Mar..

[29] Magdel-Din Hussein M., Abdel-Aziz A., Mohamed Salem H. (1980). Sulphated heteropolysaccharides from *Padina pavonia*. Phytochemistry.

[30] Mahmoud A.M., El-Derby A.M., Elsayed K.N.M., Abdella E.M. (2014). Brown seaweeds ameliorate renal alterations in mice treated with the carcinogen azoxymethane. Int. J. Pharm Pharm. Sci..

[31] Mahmoud A.M., Abdella E.M., El-Derby A.M., Abdella E.M. (2015). Protective effects of *Turbinaria ornata* and *Padina pavonia* against azoxymethane-induced colon carcinogenesis through modulation of ppar gamma, nf-kappab and oxidative stress. Phytother. Res..

[32] Abdella E., Mahmoud A., El-Derby A. (2016). Brown seaweeds protect against azoxymethane-induced hepatic repercussions through up-regulation of peroxisome proliferator activated receptor gamma and attenuation of oxidative stress. Pharm. Biol..

[33] Barbalace M.C., Malaguti M., Giusti L., Lucacchini A., Hrelia S., Angeloni C. (2019). Anti-inflammatory activities of marine algae in neurodegenerative diseases. Int. J. Mol. Sci..

[34] Abd El-Twab S.M., Mohamed H.M., Mahmoud A.M. (2016). Taurine and pioglitazone attenuate diabetes-induced testicular damage by abrogation of oxidative stress and up-regulation of the pituitary-gonadal axis. Can. J. Physiol. Pharm..

[35] Trinder P. (1969). Determination of glucose in blood using glucose oxidase with an alternative oxygen acceptor. Ann. Clin. Biochem..

[36] Haffner S.M. (2000). Coronary heart disease in patients with diabetes. N. Engl. J. Med..

[37] Seifter S., Dayton S., Novic B., Muntwyler E. (1950). The estimation of glycogen with the anthrone reagent. Arch. Biochem..

[38] Brandstrup N., Kirk J.E., Bruni C. (1957). The hexokinase and phosphoglucoisomerase activities of aortic and pulmonary artery tissue in individuals of various ages. J. Gerontol..

[39] Koide H., Oda T. (1959). Pathological occurrence of glucose-6-phosphatase in serum in liver diseases. Clin. Chim. Acta.

[40] Freedland R.A., Harper A.E. (1959). Metabolic adaptations in higher animals. V. The study of metabolic pathways by means of metabolic adaptations. J. Biol. Chem..

[41] Stalmans W., Hers H.G. (1975). The stimulation of liver phosphorylase b by amp, fluoride and sulfate. A technical note on the specific determination of the a and b forms of liver glycogen phosphorylase. Eur. J. Biochem..

[42] Fiske C., Subbarow Y. (1925). The colourimetric determination of phosphorus. J. Biol. Chem..

[43] Ross R., Braunwald E. (1992). The pathogenesis of atherosclerosis. Heart Disease: A Textbook of Cardiovascular Medicine.

[44] Folch J., Lees M., Sloane Stanley G.H. (1957). A simple method for the isolation and purification of total lipides from animal tissues. J. Biol. Chem..

[45] Hozayen W.G., Mahmoud A.M., Desouky E.M., El-Nahass E.-S., Soliman H.A., Farghali A.A. (2019). Cardiac and pulmonary toxicity of mesoporous silica nanoparticles is associated with excessive ros production and redox imbalance in wistar rats. Biomed. Pharmacother..

[46] Preuss H.G., Jarrell S.T., Scheckenbach R., Lieberman S., Anderson R.A. (1998). Comparative effects of chromium, vanadium and *Gymnema sylvestre* on sugar-induced blood pressure elevations in shr. J. Am. Coll. Nutr..

[47] Grisham M.B., Johnson G.G., Lancaster J.R. (1996). Quantitation of nitrate and nitrite in extracellular fluids. Methods Enzymol..

[48] Beutler E., Duron O., Kelly B.M. (1963). Improved method for the determination of blood glutathione. J. Lab. Clin. Med..

[49] Marklund S., Marklund G. (1974). Involvement of the superoxide anion radical in the autoxidation of pyrogallol and a convenient assay for superoxide dismutase. Eur. J. Biochem..

[50] Cohen G., Dembiec D., Marcus J. (1970). Measurement of catalase activity in tissue extracts. Anal. Biochem..

[51] Mahmoud A.M. (2014). Hesperidin protects against cyclophosphamide-induced hepatotoxicity by upregulation of pparγ and abrogation of oxidative stress and inflammation. Can. J. Physiol. Pharm..

[52] Livak K.J., Schmittgen T.D. (2001). Analysis of relative gene expression data using real-time quantitative pcr and the 2(-delta delta c(t)) method. Methods.

[53] Bradford M.M. (1976). A rapid and sensitive method for the quantitation of microgram quantities of protein utilizing the principle of protein-dye binding. Anal. Biochem..

[54] Pettersen E.F., Goddard T.D., Huang C.C., Couch G.S., Greenblatt D.M., Meng E.C., Ferrin T.E. (2004). Ucsf chimera—A visualization system for exploratory research and analysis. J. Comput. Chem..

[55] Nolte R.T., Wisely G.B., Westin S., Cobb J.E., Lambert M.H., Kurokawa R., Rosenfeld M.G., Willson T.M., Glass C.K., Milburn M.V. (1998). Ligand binding and co-activator assembly of the peroxisome proliferator-activated receptor-γ. Nature.

[56] Choi S.U., Yang M.C., Lee K.H., Kim K.H., Lee K.R. (2007). Lignan and terpene constituents from the aerial parts of *Saussurea pulchella*. Arch. Pharmacal Res..

[57] Häusler M., Montag A. (1989). Isolation, identification and quantitative determination of the norisoprenoid (s)-(+)-dehydrovomifoliol in honey. Z. Lebensm. -Unters. Forsch..

[58] He Z., Zhang A., Ding L., Lei X., Sun J., Zhang L. (2010). Chemical composition of the green alga codium divaricatum holmes. Fitoterapia.

[59] Park J.-H., Lee D.-G., Yeon S.-W., Kwon H.-S., Ko J.-H., Shin D.-J., Park H.-S., Kim Y.-S., Bang M.-H., Baek N.-I. (2011). Isolation of megastigmane sesquiterpenes from the silkworm (*Bombyx mori* l.) droppings and their promotion activity on ho-1 and sirt1. Arch. Pharmacal Res..

[60] Liu X., Wu Q.X., Shi Y.P. (2005). Terpenoids from the flower of *Cacalia tangutica*. J. Chin. Chem. Soc..

[61] Kwon H.C., Lee K.R. (2001). Phytochemical constituents of *Artemisia japonica* ssp. Littoricola. Arch. Pharmacal Res..

[62] Laakso M. (1993). How good a marker is insulin level for insulin resistance?. Am. J. Epidemiol..

[63] Lee Y.S., Li P., Huh J.Y., Hwang I.J., Lu M., Kim J.I., Ham M., Talukdar S., Chen A., Lu W.J. (2011). Inflammation is necessary for long-term but not short-term high-fat diet-induced insulin resistance. Diabetes.

[64] Breyer M.D., Böttinger E., Brosius F.C., Coffman T.M., Harris R.C., Heilig C.W., Sharma K. (2005). Mouse models of diabetic nephropathy. J. Am. Soc. Nephrol..

[65] Association A.D. (2014). Standards of medical care in diabetes—2014. Diabetes Care.

[66] Guex C.G., Reginato F.Z., De Jesus P.R., Brondani J.C., Lopes G.H.H., Bauermann L.F. (2019). Antidiabetic effects of *Olea europaea* l. Leaves in diabetic rats induced by high-fat diet and low-dose streptozotocin. J. Ethnopharmacol..

[67] Liao Z., Zhang J., Liu B., Yan T., Xu F., Xiao F., Wu B., Bi K., Jia Y. (2019). Polysaccharide from okra (*Abelmoschus esculentus* (l.) moench) improves antioxidant capacity via pi3k/akt pathways and nrf2 translocation in a type 2 diabetes model. Molecules.

[68] Mahmoud A.M., Germoush M.O., Elgebaly H.A., Elsayed K.N.M., Hassan S., Mousa N.M., Hussein O.E. (2014). Antidiabetic and insulin sensitizing effects of *Padina pavonia* and *Turbenaria ornata* in streptozotocin/nicotinamide diabetic rats. Asian J. Pharm Clin. Res..

[69] Nordlie R.C., Foster J.D., Lange A.J. (1999). Regulation of glucose production by the liver. Annu. Rev. Nutr..

[70] Gothandam K., Ganesan V.S., Ayyasamy T., Ramalingam S. (2019). Antioxidant potential of theaflavin ameliorates the activities of key enzymes of glucose metabolism in high fat diet and streptozotocin—Induced diabetic rats. Redox Rep..

[71] Mishra C., Khalid M.A., Fatima N., Singh B., Tripathi D., Waseem M., Mahdi A.A. (2019). Effects of citral on oxidative stress and hepatic key enzymes of glucose metabolism in streptozotocin/high-fat-diet induced diabetic dyslipidemic rats. Iran. J. Basic Med. Sci..

[72] Gupta D., Raju J., Prakash J., Baquer N.Z. (1999). Change in the lipid profile, lipogenic and related enzymes in the livers of experimental diabetic rats: Effect of insulin and vanadate. Diabetes Res. Clin. Pract..

[73] Ahmed O.M., Moneim A.A., Mahmoud A.M., Yazid I.A. (2010). Antihyperglycemic, antihyperlipidemic and antioxidant effects and the probable mechanisms of action of *Ruta graveolens* infusion and rutin in nicotinamide-streptozotocin-induced diabetic rats. Diabetol. Croat..

[74] Pilkis S.J., Claus T.H. (1991). Hepatic gluconeogenesis/glycolysis: Regulation and structure/function relationships of substrate cycle enzymes. Annu. Rev. Nutr..

[75] Roden M., Bernroider E. (2003). Hepatic glucose metabolism in humans—Its role in health and disease. Best Pract. Res. Clin. Endocrinol. Metab..

[76] Rui L. (2014). Energy metabolism in the liver. Compr. Physiol..

[77] Golden S., Wals P.A., Okajima F., Katz J. (1979). Glycogen synthesis by hepatocytes from diabetic rats. Biochem. J..

[78] Postic C., Dentin R., Girard J. (2004). Role of the liver in the control of carbohydrate and lipid homeostasis. Diabetes Metab..

[79] Reaven G.M. (2005). Compensatory hyperinsulinemia and the development of an atherogenic lipoprotein profile: The price paid to maintain glucose homeostasis in insulin-resistant individuals. Endocrinol. Metab. Clin. N. Am..

[80] Ahmed O.M., Mahmoud A.M., Abdel-Moneim A., Ashour M.B. (2012). Antidiabetic effects of hesperidin and naringin in type 2 diabetic rats. Diabetol. Croat..

[81] Betteridge D.J. (2007). Effects of pioglitazone on lipid and lipoprotein metabolism. Diabetes Obes. Metab..

[82] Kazumi T., Hirano T., Odaka H., Ebara T., Amano N., Hozumi T., Ishida Y., Yoshino G. (1996). Vldl triglyceride kinetics in wistar fatty rats, an animal model of niddm: Effects of dietary fructose alone or in combination with pioglitazone. Diabetes.

[83] Levinthal G.N., Tavill A.S. (1999). Liver disease and diabetes mellitus. Clin. Diabetes.

[84] Nannipieri M., Gonzales C., Baldi S., Posadas R., Williams K., Haffner S.M., Stern M.P., Ferrannini E. (2005). Liver enzymes, the metabolic syndrome, and incident diabetes: The mexico city diabetes study. Diabetes Care.

[85] Mohamed J., Nazratun Nafizah A.H., Zariyantey A.H., Budin S.B. (2016). Mechanisms of diabetes-induced liver damage: The role of oxidative stress and inflammation. Sultan Qaboos Univ. Med. J..

[86] Mahmoud A.M., Xiao J. (2017). Exercise amaliorates metabolic disturbances and oxidative stress in diabetic cardiomyopathy: Possible underlying mechanisms. Exercise for Cardiovascular Disease Prevention and Treatment: From Molecular to Clinical, Part 1.

[87] Aladaileh S.H., Abukhalil M.H., Saghir S.A.M., Hanieh H., Alfwuaires M.A., Almaiman A.A., Bin-Jumah M., Mahmoud A.M. (2019). Galangin activates nrf2 signaling and attenuates oxidative damage, inflammation, and apoptosis in a rat model of cyclophosphamide-induced hepatotoxicity. Biomolecules.

[88] Ranneh Y., Akim A.M., Hamid H.A., Khazaai H., Fadel A., Mahmoud A.M. (2019). Stingless bee honey protects against lipopolysaccharide induced-chronic subclinical systemic inflammation and oxidative stress by modulating nrf2, nf-κb and p38 mapk. Nutr. Metab..

[89] Elgebaly H.A., Mosa N.M., Allach M., El-Massry K.F., El-Ghorab A.H., Al Hroob A.M., Mahmoud A.M. (2018). Olive oil and leaf extract prevent fluoxetine-induced hepatotoxicity by attenuating oxidative stress, inflammation and apoptosis. Biomed. Pharm..

[90] Mahmoud A.M., Hussein O.E., Abd El-Twab S.M., Hozayen W.G. (2019). Ferulic acid protects against methotrexate nephrotoxicity via activation of nrf2/are/ho-1 signaling and pparγ, and suppression of nf-κb/nlrp3 inflammasome axis. Food Funct..

[91] Abd El-Twab S.M., Hussein O.E., Hozayen W.G., Bin-Jumah M., Mahmoud A.M. (2019). Chicoric acid prevents methotrexate-induced kidney injury by suppressing nf-κb/nlrp3 inflammasome activation and up-regulating nrf2/are/ho-1 signaling. Inflamm. Res..

[92] Kersten S., Desvergne B., Wahli W. (2000). Roles of ppars in health and disease. Nature.

[93] Remels A.H., Langen R.C., Gosker H.R., Russell A.P., Spaapen F., Voncken J.W., Schrauwen P., Schols A.M. (2009). Ppargamma inhibits nf-kappab-dependent transcriptional activation in skeletal muscle. Am. J. Physiol. Endocrinol. Metab..

[94] Okuno Y., Matsuda M., Miyata Y., Fukuhara A., Komuro R., Shimabukuro M., Shimomura I. (2010). Human catalase gene is regulated by peroxisome proliferator activated receptor-gamma through a response element distinct from that of mouse. Endocr. J..

[95] Hwang J., Kleinhenz D.J., Lassegue B., Griendling K.K., Dikalov S., Hart C.M. (2005). Peroxisome proliferator-activated receptor-gamma ligands regulate endothelial membrane superoxide production. Am. J. Physiol. Cell Physiol..

[96] Park S.H., Choi E., Kim S., Kim D.S., Kim J.H., Chang S., Choi J.S., Park K.J., Roh K.-B., Lee J. (2018). Oxidative stress-protective and anti-melanogenic effects of loliolide and ethanol extract from fresh water green algae, prasiola japonica. Int. J. Mol. Sci..

